# Supplementary material for: Localising enzymes to biomolecular condensates increases their accumulation and benefits engineered metabolic pathway performance in Nicotiana benthamiana
Source: Plant Biotechnol J. 2025 Apr 9;24(1):171–86. doi: 10.1111/pbi.70082 (PMC12854901; doi:10.1111/pbi.70082)
Supplement: Supplementary file 1 — Figure S1 Further characterisation of synthetic biomolecular condensates in N. benthamiana. Figure S2 Alignment between MdCMS and closely related methylthioalkylmalate synthase 3 (MAM3) from A. thaliana used to determine whether MdCMS contains a chloroplast transit peptide. Figure S3 Further characterisation of PHB‐functionalised condensates. Figure S4 Absence of co‐localisation of mClover3 and Nile red signal in representative images of N. benthamiana leaf transiently expressing the RGG‐containing scaffold (+ RGG) and the PHB‐enzyme client (+ PHB) constructs 3 dpi. Figure S5 Semi‐quantitative analysis of scaffold and client levels in each image, shown here for β‐ketothiolase (PhbA) and acetoacetyl‐CoA reductase (PhbB). [file PBI-24-171-s004.docx]

**
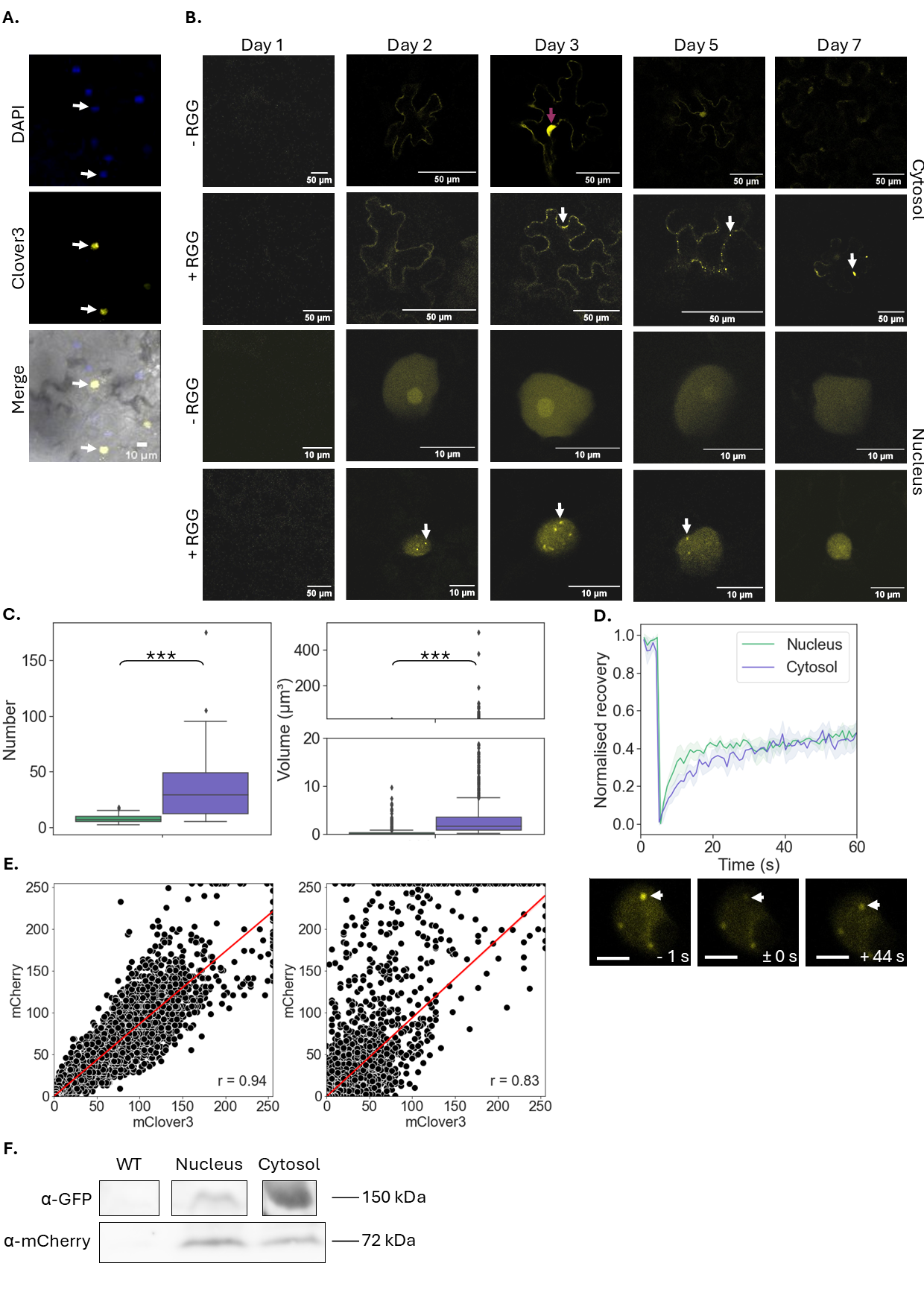
**

**Figure S1: Further characterisation of synthetic biomolecular condensates in *N. benthamiana*.** (A) DAPI-staining co-localises with mClover3 signal when an NLS is included, indicating that mClover3 gets targeted to the nucleus. (B) Timecourse of expression over 7 dpi in nuclei and cytosol of tissue infiltrated with the RGG-containing or no-RGG-containing version of the scaffold. mClover3 signal is shown. White arrows indicate condensates. Purple arrow indicates a nucleus; the presence of signal in the nuclei of cells overexpressing a fluorescent protein is common. (C) A comparison of the number per cell (left) and volume (right) of cytosolic (blue) and nuclear (green) biomolecular condensates. The distribution is plotted using a box and whisker plot, where the box shows the mean and the interquartile range. The whiskers extend to values above or below 1.5 × the interquartile range. Points either above or below this value are shown as diamonds. ‘***’ represents a *P*-value < 0.001, ‘**’ a *P*-value < 0.01, ‘*’ a *P*-value < 0.05, or ‘n.s.’ a *P*-value > 0.05 (Mann-Whitney *U*). *n* = 21-54 (number) or 400-880 (volume). (D) Normalised FRAP curves for nuclear and cytosolic biomolecular condensates (top), and example of biomolecular condensate bleaching in a nucleus (bottom). *n* = 4-5. Arrow indicates bleached biomolecular condensate. Pre (-) or post (+) bleaching time indicated. Scale bar is 10 μm and shaded area represents SEM. (E) The relationship between mCherry and mClover3 signal in a leaf expressing the nuclear (left) or cytosolic (right) version of the scaffold. Shaded area represents SEM, and the Pearsons correlation coefficient (*r*) is shown in the bottom-right corner. (F) Western blot for the detection of mClover3 (α-GFP) and mCherry in crude leaf extract of agroinfiltrated *N. benthamiana* leaves 5 dpi.

**Figure S2: Alignment between *Md*CMS and closely related methylthioalkylmalate synthase 3 (MAM3) from *A. thaliana* used to determine whether *Md*CMS contains a chloroplast transit peptide.** The alignment analysis was performed using EMBOSS Stretcher from EMBL-EBI (Madeira *et al.*, 2022). MAM3 is known to be chloroplast localised and has a long unstructured region on its N-terminus which is predicted to encode a chloroplast transit peptide (see UniProt accession Q9FN52). In the predicted structure for *Md*CMS, the N-terminus is also unstructured when analysed using AlphaFold (Jumper *et al*., 2021) and so we hypothesized that *Md*CMS also has an N-terminal chloroplast transit peptide. TargetP-2.0 (Almagro Armenteros *et al*., 2019) was used to predict the presence of a N-terminal chloroplast transit peptide with high probability, including a putative cleavage site, in the *Md*CMS protein sequence. N-terminal unstructured region indicated in orange and predicted cleavage sites shown using a red arrow.

**
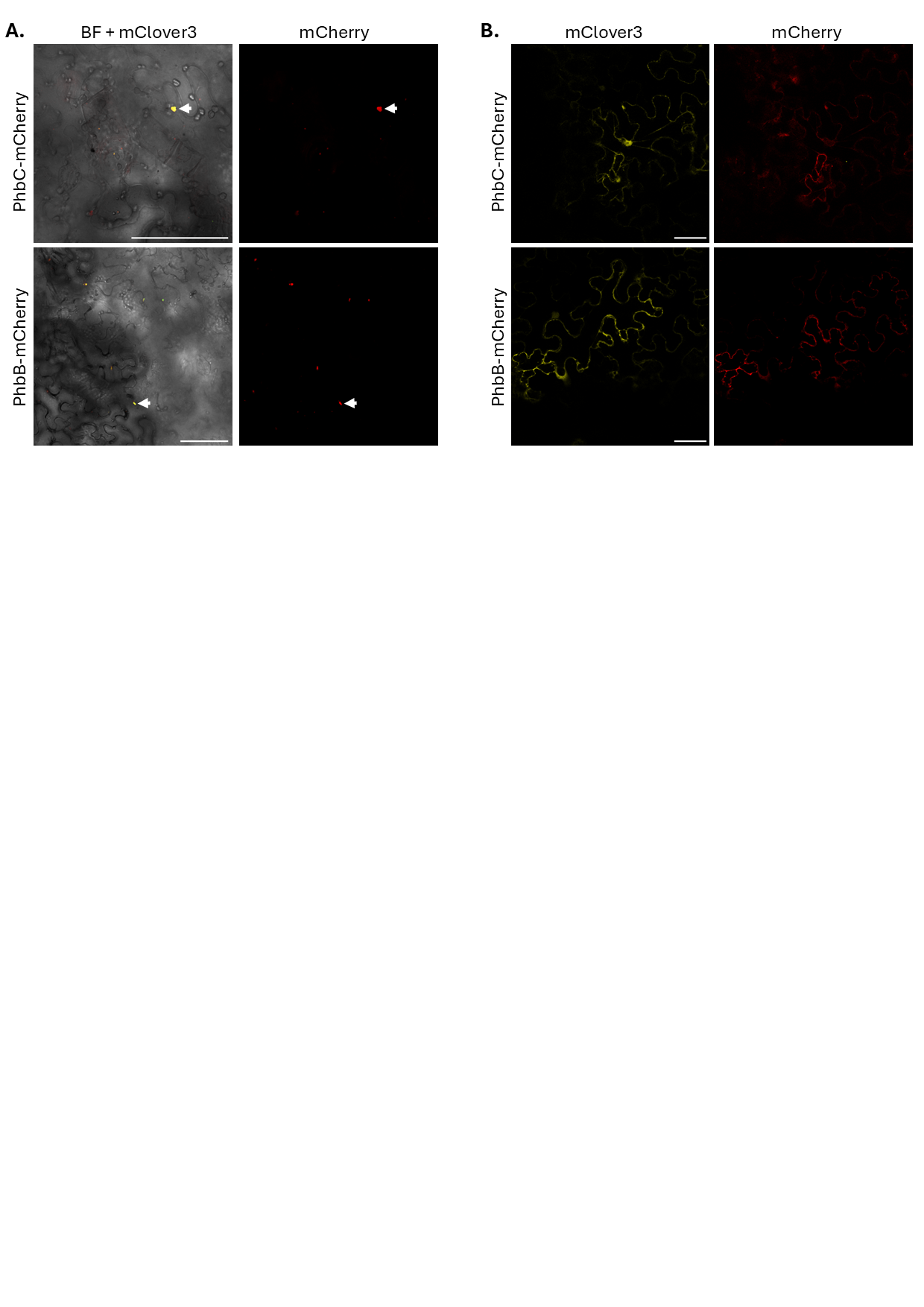
**

**Figure S3: Further characterisation of PHB-functionalised condensates.** (A) Co-localisation of mClover3 and mCherry-fused acetoacetyl-CoA reductase (PhbB), and PHB synthase (PhbC) in biomolecular condensates. White arrow indicates a condensate. (B) The removal of the RGG domain ablates biomolecular condensate formation and results in diffuse mClover3 and mCherry cytosolic signal. Scale bar in both panels is 100 μm.

**
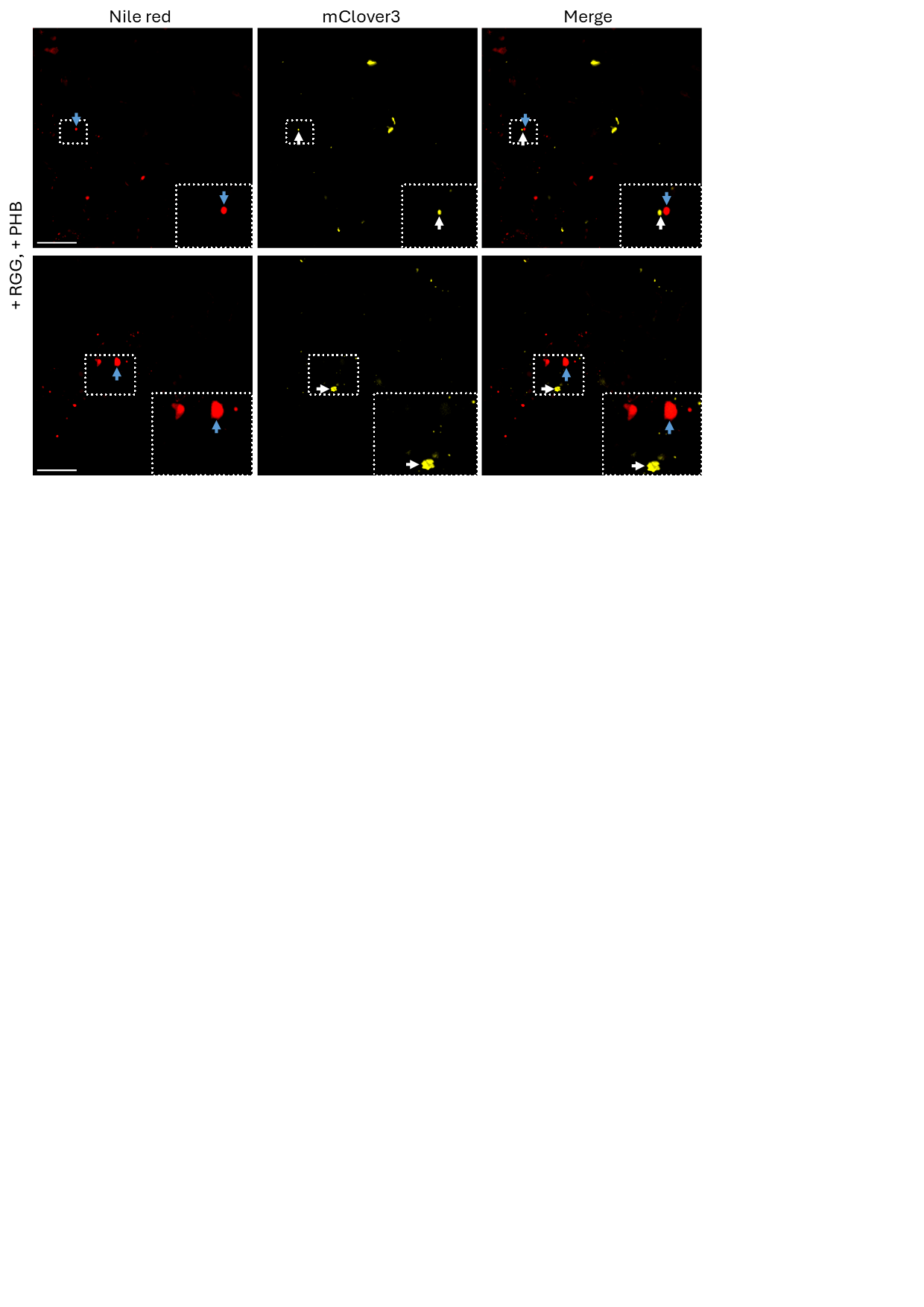
**

**Figure S4: Absence of co-localisation of mClover3 and Nile red signal in representative images of *N. benthamiana* leaf transiently expressing the RGG-containing scaffold (+ RGG) and the PHB-enzyme client (+ PHB) constructs 3 dpi**. White arrows indicate a condensate, and blue arrows indicate a probable lipid droplet. Scale bar is 50 μm.

**
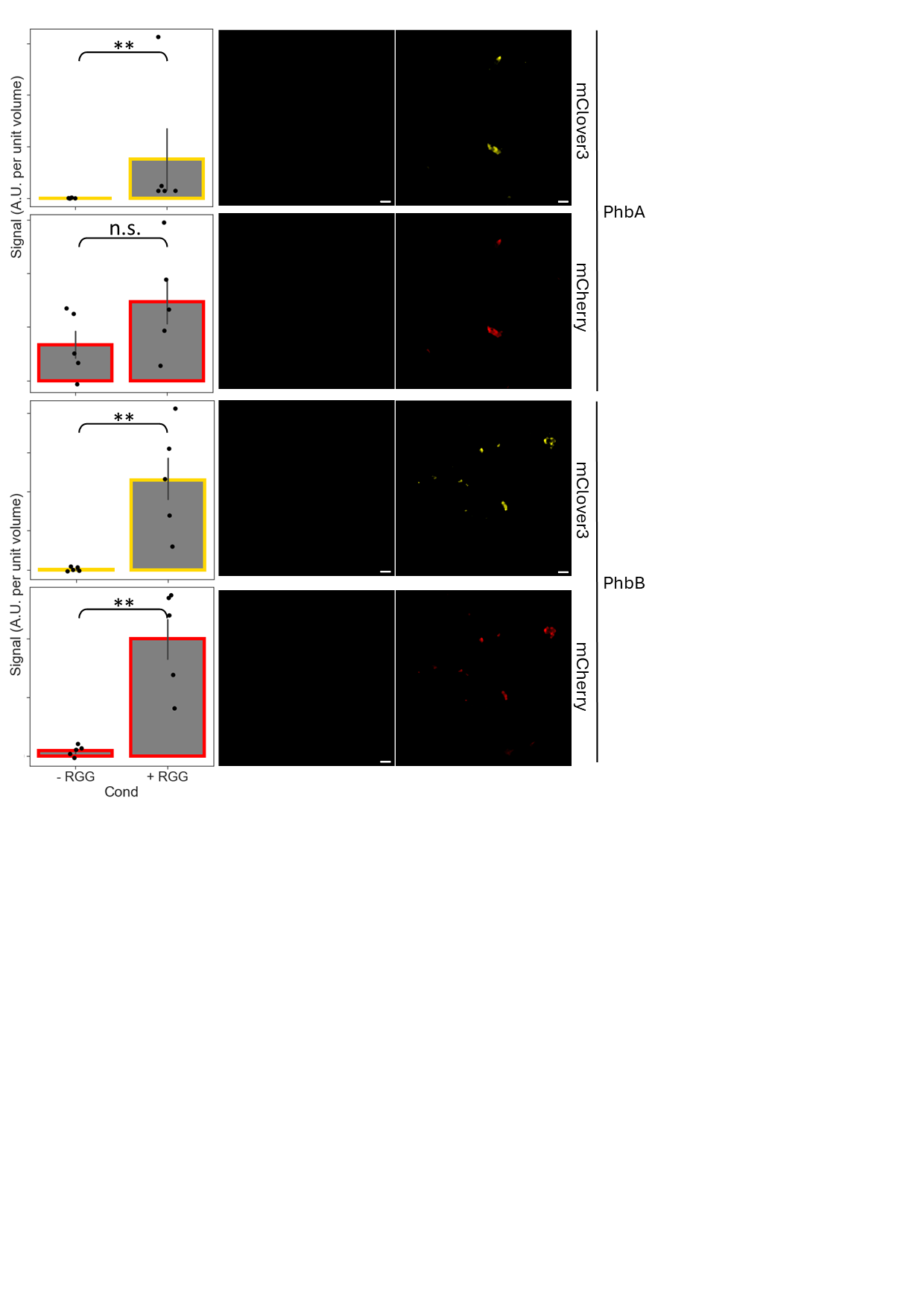
**

**Figure S5: Semi-quantitative analysis of scaffold and client levels in each image, shown here for β-ketothiolase (PhbA) and acetoacetyl-CoA reductase (PhbB).** Error bars represent SEM; ‘***’ represents a *P*-value < 0.001, ‘**’ a *P*-value < 0.01, ‘*’ a *P*-value < 0.05, or ‘n.s.’ a *P*-value > 0.05 (Mann-Whitney *U*). *n* = 5.

**Supplementary References**

Almagro Armenteros, J. J., Salvatore, M., Emanuelsson, O., Winther, O., von Heijne, G., Elofsson, A. and Nielsen, H. (2019). Detecting sequence signals in targeting peptides using deep learning*. Life Sci. Alliance,* 2, e201900429.

Jumper, J., Evans, R., Pritzel, A., Green, T., Figurnov, M., Ronneberger, O., Tunyasuvunakool, K. *et al*. (2021). Highly accurate protein structure prediction with AlphaFold. *Nature,* 596, 583-589.

Madeira, F., Pearce, M., Tivey, A. R. N., Basutkar, P., Lee, J., Edbali, O., Madhusoodanan, N. *et al*. (2022). Search and sequence analysis tools services from EMBL-EBI in 2022. *Nucleic Acids Res.* 50(W1), W276-W279.
