# Supplementary material for: Localising enzymes to biomolecular condensates increases their accumulation and benefits engineered metabolic pathway performance in Nicotiana benthamiana
Source: Plant Biotechnol J. 2025 Apr 9;24(1):171–86. doi: 10.1111/pbi.70082 (PMC12854901; doi:10.1111/pbi.70082)
Supplement: Supplementary file 2 — Table S1 Gene parts used in this study. [file PBI-24-171-s002.docx]

**Table S1: Gene parts used in this study.**

| Name | Description | Sequence source or protein sequence |
| --- | --- | --- |
| PhbA | Coding sequence for β-ketothiolase from *R. eutropha* | Genbank accession number FJ897461.1 |
| PhbB | Coding sequence for acetoacetyl-CoA reductase from *R. eutropha* | Genbank accession number FJ897462.1 |
| PhbC | Coding sequence for PHB synthase from *R. eutropha* | Genbank accession number MH558939.1 |
| P2A | Self-cleaving 2A peptide from porcine teschovirus-1 2A (Szymczak-Workman *et al*., 2012). 2A peptides are 18-22 amino acid viral oligopeptides which mediate the cleavage of polypeptides during translation in eukaryotic systems, by the ribosome skipping of the formation of a glycyl-prolyl peptide bond at its C-terminus (Donnelly *et al*., 2001). | (Wang *et al*., 2015) |
| T2A | Self-cleaving 2A peptide from *Thosea asigna* virus 2A (Szymczak-Workman *et al.*, 2012). | (Wang *et al*., 2015) |
| InteinF2A | Self-cleaving fusion protein domain composed of a *Ssp* DnaE mini-intein variant engineered for hyper-N-terminal autocleavage which is covalently linked to the F2A peptide (self-cleaving 2A peptide from foot-and-mouth disease virus) (Zhang *et al*., 2017). | (Zhang *et al*., 2017) |
| GSG linker | A glycine-serine-glycine (GSG) linker fused to the N-terminus of 2A peptides which can improve cleavage efficiency. | (Wang *et al*., 2015) |
| RGG | IDR from the *C. elegans* protein LAF-1 | (Schuster *et al*., 2020) |
| SYNZIP1 and SYNZIP2 | Heterospecific synthetic coiled-coil peptide pair | (Thompson *et al*., 2012) |
| mCherry | Monomeric red fluorescent protein | Genbank accession number AY678264.1 |
| mClover3 | Monomeric green/yellow fluorescent protein | Genbank accession number ATE88096.1 |
| *Md*CMS | Citramalate synthase enzyme MdCMS_1 from *Malus x domestica*. | (Sugimoto *et al*., 2021) |
| His | Polyhistidine tag enabling purification of the constructs using Ni-affinity purification. | HHHHHHHHHH |
| Myc | Short protein tag derived from the c-myc gene product and used to detect proteins in Western blotting | EQKLISEEDL |
| HA | Short protein tag derived from the human influenza hemagglutinin surface glycoprotein and used to detect proteins in Western blotting | YPYDVPDYA |

Donnelly, M. L. L., Hughes, L. E., Luke, G., Mendoza, H., ten Dam, E., Gani, D. and Ryan, M. D. (2001) The 'cleavage' activities of foot-and-mouth disease virus 2A site-directed mutants and naturally occurring '2A-like' sequences. *J. Gen. Virol*. 82, 1027-1041.

Schuster, B. S., Dignon, G. L., Tang, W. S., Kelley, F. M., Ranganath, A. K., Jahnke, C. N., Simpkins, A. G. et al. (2020) Identifying sequence perturbations to an intrinsically disordered protein that determine its phase-separation behavior. *Proc. Natl Acad. Sci. USA*, 117, 11421-11431.

Szymczak-Workman, A. L., Vignali, K. M, and Vignali D. A. A. (2012) Design and construction of 2A peptide-linked multicistronic vectors. *Cold Spring Harb. Protoc.* 2012, 199-204.

Wang, Y. C., Wang, Y., Wang, F., Wang, R., Zhao, P, and Xia, Q. (2015) 2A self-cleaving peptide-based multi-gene expression system in the silkworm *Bombyx mori*. *Sci. Rep.* 5, 16273.
