# Supplementary material for: Localising enzymes to biomolecular condensates increases their accumulation and benefits engineered metabolic pathway performance in Nicotiana benthamiana
Source: Plant Biotechnol J. 2025 Apr 9;24(1):171–86. doi: 10.1111/pbi.70082 (PMC12854901; doi:10.1111/pbi.70082)
Supplement: Supplementary file 3 — Table S2 Primers used in this study. [file PBI-24-171-s001.docx]

**Table S2: Primers used in this study.**

| Name | Sequence | Description |
| --- | --- | --- |
| Sequencing | | |
| AB_seq1 | cggcaggatgcttaacataag | Sequencing primer for condensate-forming constructs, binding in mCherry |
| AB_seq2 | tcttgctcgcctctatcatgc | Sequencing primer for condensate-forming constructs, binding in IntF2A |
| AB_seq3 | cagcagcagtaacaaactcg | Sequencing primer for condensate-forming constructs, binding in mClover3 |
| AB_seq4 | gttcctcacctttgctcac |  |
| M13F | gtaaaacgacggccagt | In-house sequencing primer M13F, binding in cloning vector backbone |
| M13R | caggaaacagctatgac | In-house sequencing primer M13R, binding in cloning vector backbone |
| Gibson assembly | | |
| GA1F | cgaacaatgaacccagctttcttgtacaaag | Primers used for the assembly of GA2.1 (mClo3R and RGGF) and GA2.1noNLS (mClo3RnoNLS and RGGFnoNLS) from gene fragments synthesized by TWIST and pmClo3_FL (mClover3) |
| GA1R | ctttgctcacaccgtctccaccattgtc |  |
| mClo3F | tggagacggtgtgagcaaaggtgaggaac |  |
| mClo3R | gattgctttccactttacgctttttcttcg |  |
| mClo3RnoNLS | gattgctttctttgtaaagttcgtccatacc |  |
| RGGF | gcgtaaagtggaaagcaatcagagcaataac |  |
| RGGFnoNLS | actttacaaagaaagcaatcagagcaataac |  |
| RGGR | gcccgtccccaccattgtcccttcgatc |  |
| mCheF | ggacaatggtggggacgggcatcaccac |  |
| mCheR | aaagctgggttcattgttcgtgagaggccac |  |
| GA3_1F | tgacgataaggtgagcaaaggtgaggaac | Primers used for the assembly of GA3 (GA3_1R and GA3_2F) and GA3noNLS (GA3_1noNLSR and GA3_2noNLSF) from GA2.1 and GA2.1noNLS |
| GA3_1R | gatgatgatgcactttacgctttttcttcgg |  |
| GA3_1noNLSR | gatgatgatgtttgtaaagttcgtccatacc |  |
| GA3_2F | gcgtaaagtgcatcatcatcatcatcactgc |  |
| GA3_2noNLSF | actttacaaacatcatcatcatcatcactgc |  |
| GA3_2R | ctttgctcaccttatcgtcatcatctttataatcttc |  |
| MdCMS_fwd | tcacgttagcatggccttctcagcagaaaatc | Primers used for the assembly of GA2.1noNLS_MdCMS (MdCMS_fwd and BB_rev) and GA2.1noNLS_MdCMSnoChl (MdCMSnoChl_fwd and BBnoChl_rev) |
| MdCMSnoChl_fwd | tcacgttagccccactcaccccgaatac |  |
| MdCMS_rev | tatgagatgtgatgtccaacttgatgttgac |  |
| BB_fwd | gttggacatcacatctcataacgaggattac |  |
| BB_rev | agaaggccatgctaacgtgatgatgatg |  |
| BBnoChl_rev | ggtgagtggggctaacgtgatgatgatg |  |
| RGG_mClover3_fwd | tgaggaacttttcactggggttgtgcctatc | Primers used for the assembly of SYNZIP1_RGG_mClo3 from GA2.1noNLS |
| RGG_mClover3_rev | gctgggttcaatgatgatgatggtggtgatgc |  |
| mClover3_RGG_BB_fwd | tcatcatcattgaacccagctttcttgtac |  |
| mClover3_RGG_BB_rev | ccccagtgaaaagttcctcacctttgctc |  |
| mClo_fwd | tgacgataaggtgagcaaaggtgaggaac | Primers used for assembly of SYNZIP1_mClo3 from SYNZIP1_RGG_mClo3 |
| mClo_rev | ggtggtgatgtttgtaaagttcgtccatacc |  |
| ZIP1BB_fwd | actttacaaacatcaccaccatcatcatc |  |
| ZIP1BB_rev | ctttgctcaccttatcgtcatcatctttataatc |  |
| SYNZIP2_PhbB2546_fwd | taggtggaggctcagtgatgataagtctg | Primers used for the assembly of SYNZIP2_PhbABC from gene fragments synthesized by TWIST |
| SYNZIP2_PhbB2546_rev | cctagtaatacctgcattattaatcaagac |  |
| SYNZIP2_PhbB2547_fwd | ataatgcaggtattactagggacgttgtctttc |  |
| SYNZIP2_PhbB2547_rev | catcactgagcctccacctagcctcaaataatg |  |
| BB1_fwd | ctcgacataagcaaaagatagtggcacc | Primers used for the assembly of SYNZIP2_PhbAmCherry_BC (BB1PhbA_rev, mCherryLinkerPhbA_fwd, BB2PhbA_fwd), SYNZIP2_PhbABmCherry_C (BB1PhbB_rev, mCherryLinkerPhbB_fwd, BB2PhbB_fwd), and SYNZIP2_PhbABCmCherry (BB1PhbC_rev, mCherryLinkerPhbC_fwd, BB2PhbC_fwd) from pK7WG2_MdCMS_mC_SpC and SYNZIP2_PhbABC |
| BB1PhbA_rev | ccgtagctccgtgatgatgttgttcgtg |  |
| BB1PhbB_rev | ccgtagctccatggtggtgttgttcgtg |  |
| BB1PhbC_rev | ccgtagctccagccttggccttaacgtatc |  |
| mCherryLinkerPhbA_fwd | acatcatcacggagctacggccagtgct |  |
| mCherryLinkerPhbB_fwd | acaccaccatggagctacggccagtgct |  |
| mCherryLinkerPhbC_fwd | ggccaaggctggagctacggccagtgct |  |
| mCherryLinkerPhbA_rev | caacgtcggtatcgtagtacgacattgcgcc |  |
| mCherryLinkerPhbB_rev | ccttatagtcatcgtagtacgacattgcgcc |  |
| mCherryLinkerPhbC_rev | cgtaaggataatcgtagtacgacattgcgcc |  |
| BB2PhbA_fwd | gtactacgataccgacgttgtgattgtc |  |
| BB2PhbB_fwd | gtactacgatgactataaggatgatgacgataaaatg |  |
| BB2PhbC_rev | gtactacgattatccttacgatgttccgg |  |
| BB2_rev | tatcttttgcttatgtcgagcctccgtc |  |
