# Supplementary material for: Localising enzymes to biomolecular condensates increases their accumulation and benefits engineered metabolic pathway performance in Nicotiana benthamiana
Source: Plant Biotechnol J. 2025 Apr 9;24(1):171–86. doi: 10.1111/pbi.70082 (PMC12854901; doi:10.1111/pbi.70082)
Supplement: Supplementary file 4 — Table S3 Plasmids used in this study. PhbA: β‐ketothiolase, PhbB: acetoacetyl‐CoA reductase, PhbC: PHB synthase. [file PBI-24-171-s003.docx]

**Table S3: Plasmids used in this study.** PhbA: β-ketothiolase, PhbB: acetoacetyl-CoA reductase, PhbC: PHB synthase.

| Name | Description | Source |
| --- | --- | --- |
| pK7WG2 | Gateway expression vector for overexpression in plants using the Cauliflower Mosaic Virus 35S promoter (p35S). Carries a kanamycin resistance gene. | Gift from Francesco Licausi |
| GA2.1 | Cloning vector containing the cassette coding for the RGG-based scaffold fused to mClover3 and SYNZIP1 for the formation of biomolecular condensates, and the client mCherry-SYNZIP2 separated by the InteinF2A viral self-cleaving peptide. Nuclear localized. | This study |
| GA3 | As GA2.1 but with RGG domains removed. | This study |
| GA2.1noNLS | As GA2.1 but with nuclear localization signal (NLS) removed. | This study |
| GA3noNLS | As GA3 but with NLS removed. | This study |
| pK7_GA2.1 | As GA2.1 but in the pK7WG2 expression vector. | This study |
| pK7_GA3 | As GA3 but in the pK7WG2 expression vector. | This study |
| pK7_GA2.1noNLS | As GA2.1noNLS but in the pK7WG2 expression vector. | This study |
| pK7_GA3noNLS | As GA3noNLS but in the pK7WG2 expression vector. | This study |
| pmClo3_FL | Source of mClover3 coding sequence | Gift from Francesco Licausi |
| pK7WG2_MdCMS_mC_SpC | Source of *Md*CMS coding sequence | Gift from András Sándor |
| GA2.1noNLS_MDCMSnoChl | As GA2.1noNLS but with *Md*CMS added upstream and in frame with mCherry. Putative chloroplast localization signal removed from *Md*CMS. | This study |
| pK7_GA2.1noNLS_MDCMSnoChl | As GA2.1noNLS_MdCMSnoChl but in pK7WG2 expression vector | This study |
| SYNZIP1_RGG_mClo3 | Cloning vector containing the cassette coding for the RGG-based scaffold fused to mClover3 and SYNZIP1 for the formation of biomolecular condensates. | This study |
| SYNZIP2_PhbABC | Cloning vector containing the cassette coding for PhbA-SYNZIP2, PHB-SYNZIP2, and PhbC-SYNZIP2 fusion proteins separated by the InteinF2A viral self-cleaving peptide. | This study |
| SYNZIP2_PhbAmCherry_BC | As SYNZIP2_PhbABC but with mCherry fused to PhbA. | This study |
| SYNZIP2_PhbABmCherry_C | As SYNZIP2_PhbABC but with mCherry fused to PhbB. | This study |
| SYNZIP2_PhbABCmCherry | As SYNZIP2_PhbABC but with mCherry fused to PhbC. | This study |
| pK7_SYNZIP1_RGG_mClo3 | As SYNZIP1_RGG_mClo3 but in the pK7WG2 expression vector | This study |
| pK7_SYNZIP2_PhbABC | As SYNZIP2_PhbABC but in the pK7WG2 expression vector. | This study |
| pK7_SYNZIP2_PhbAmCherry_BC | As SYNZIP2_PhbAmCherry_BC but in the pK7WG2 expression vector. | This study |
| pK7_SYNZIP2_PhbABmCherry_C | As SYNZIP2_PhbABmCherry_C but in the pK7WG2 expression vector. | This study |
| pK7_SYNZIP2_PhbABCmCherry | As SYNZIP2_PhbABCmCherry but in the pK7WG2 expression vector. | This study |
